# How Can We Advance Integrative Biology Research in Animal Science in 21st Century? Experience at University of Ljubljana from 2002 to 2022

**DOI:** 10.1089/omi.2022.0133

**Published:** 2022-11-14

**Authors:** Tanja Kunej, Simon Horvat, Janez Salobir, Blaž Stres, Špela Mikec, Tomaž Accetto, Gorazd Avguštin, Bojana Bogovič Matijašić, Angela Cividini, Andreja Čanžek Majhenič, Marko Čepon, Leon Deutsch, Ida Djurdjevič, Emil Erjavec, Gregor Gorjanc, Antonija Holcman, Dušanka Jordan, Luka Juvančič, Stane Kavčič, Ajda Kermauner, Marija Klopčič, Tina Kocjančič, Milena Kovač, Aleš Kuhar, Andrej Lavrenčič, Jakob Leskovec, Alenka Levart, Špela Malovrh, Romana Marinšek-Logar, Petra Mohar Lorbeg, Mojca Narat, Tanja Obermajer, Diana Paveljšek, Tatjana Pirman, Klemen Potočnik, Ilona Rac, Vida Rezar, Irena Rogelj, Mojca Simčič, Aleš Snoj, Simona Sušnik Bajec, Tanja Šumrada, Dušan Terčič, Primož Treven, Maša Vodovnik, Manja Zupan Šemrov, Jaka Žgajnar, Silvester Žgur, Peter Dovč

**Affiliations:** ^1^ Department of Animal Science, University of Ljubljana, Biotechnical Faculty, Slovenia.; ^2^ Department of Microbiology, University of Ljubljana, Biotechnical Faculty, Slovenia.; ^3^ The Roslin Institute and Royal (Dick) School of Veterinary Studies, University of Edinburgh, Edinburgh, United Kingdom.

**Keywords:** animal science, integrative biology, systems science, ecology, COVID-19

## Abstract

In this perspective analysis, we strive to answer the following question: how can we advance integrative biology research in the 21st century with lessons from animal science? At the University of Ljubljana, Biotechnical Faculty, Department of Animal Science, we share here our three lessons learned in the two decades from 2002 to 2022 that we believe could inform integrative biology, systems science, and animal science scholarship in other countries and geographies. Cultivating multiomics knowledge through a conceptual lens of integrative biology is crucial for life sciences research that can stand the test of diverse biological, clinical, and ecological contexts. Moreover, in an era of the current COVID-19 pandemic, animal nutrition and animal science, and the study of their interactions with human health (and vice versa) through integrative biology approaches hold enormous prospects and significance for systems medicine and ecosystem health.

## Perspective

Integrative biology has various definitions depending on different application contexts and research objectives, for example. In the case of systems science, multiomics integration of diverse and complementary strands of data and insights from genomics to metabolomics to phenomics is of great interest to integrative biology. Although integration comes to the fore in this context, it is also important to sustain a highly diverse portfolio of research in omics science and biology that can provide the required inputs for integrative biology knowledge outputs. In medicine, many such multiomics research studies have already uncovered novel genes, diagnostic biomarkers, and therapeutics (Lin et al, 2022), whereas in animal science we still lag behind in this area.

**FIG. 1.**
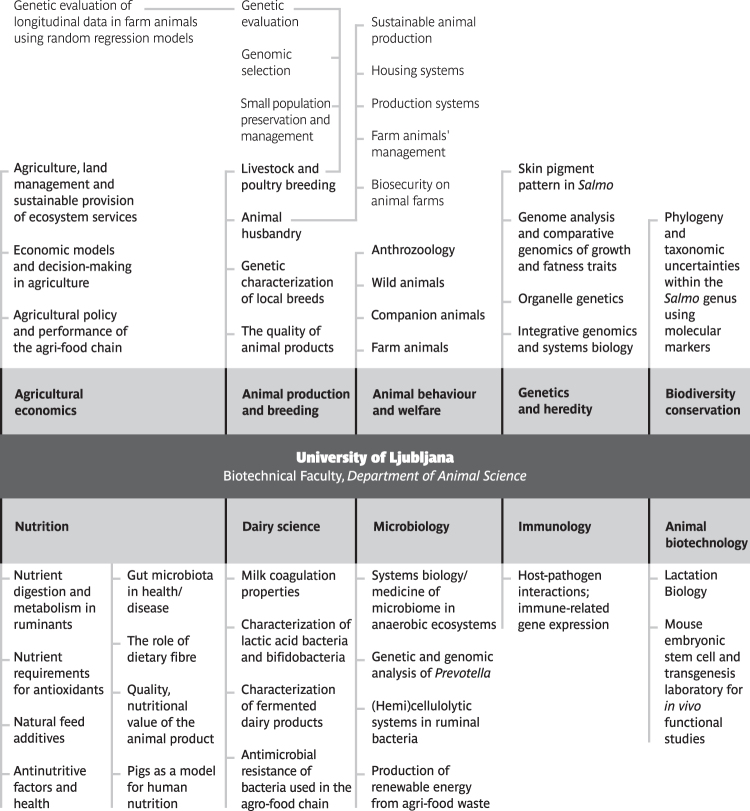
Research fields and subfields of University of Ljubljana, Biotechnical Faculty, Department of Animal Science.

In the case of animal sciences, integrative biology is one of the main sustaining pillars and drivers of this field of scholarly inquiry. At the University of Ljubljana, Biotechnical Faculty, Department of Animal Science, we share here our three lessons learned in the two decades from 2002 to 2022 that we believe would benefit integrative biology worldwide (Fig. 1). Our goal in this perspective article, based on some of the most pressing and salient priorities in the field and the important lessons learned in animal science, is to answer the following question: how can we advance integrative biology research in the 21st century with lessons from animal science?

First, genetics is a central driving force supporting the development of animal genetic resources. During the past 20 years, our research group shifted the research program, which was originally based on quantitative genetics and cytogenetics, to a conceptual lens that is more based on integrative biology. This allowed us to work across technology and disciplinary knowledge silos, and thereby molecularly tackle phenotypic traits of importance for integrative biology such as growth, fat deposition, and lactation (Ogorevc et al, 2009). We also developed transgenic and selection mouse models to study the regulation of fat deposition and identify causal genes of complex traits (Morton et al, 2016).

This type of integrative omics research that uncovers novel causal genes and pathways rather than gene and pathway associations is essential if we want to understand underlying functional mechanisms and apply them in medicine and agriculture (Zhang et al, 2022). Moreover, rapid development of functional annotation of animal genomes is expected to reveal a complex genetic base of impressive phenotypic variation in domestic animal species (Chamberlain et al, 2021).

Second, animal nutrition has critical implications for the inter-relationships among animals, nutrient requirements, feeds, bioactive and antinutritional plant compounds, health and productivity, quality and nutritional value of animal products, and the environment. Antioxidant requirements in hot climates and under different nutritional and health conditions are poorly understood. Our results, obtained using various classical methods and contemporary genetic markers of oxidative status, collectively indicate that the interactions between different antioxidants, health, and environment must be considered for integrative biology theory and applications (Korošec et al, 2017; Pečjak et al, 2022).

In a context of the current COVID-19 pandemic, animal nutrition and animal science and the study of their interactions with human health (and vice versa) through integrative biology approaches hold enormous prospects and significance in systems science and planetary health, not to mention in modeling and controlling of pandemics in the future (Kretzschmar et al, 2022; Wang et al, 2021).

Third, animal microbiome is being continuously recognized as one of the key drivers of animal development and health. As already noted, animal health is also pertinent for planetary health, human health, and zoonotic infections such as COVID-19. Methodological and conceptual developments over the past decade enabled us to integrate data at the levels of microbial taxonomy, functional genes, transcripts, enzymatic reactions, and metabolic pathways in ways that advance a systems biology framework.

We interrogated biological systems' states over different time and size scales in relation to environmental parameters. It is noteworthy that the microbiome is only one subsystem among many in the whole animal system that interacts top-down and bottom-up across the biological hierarchy and all omic layers (Stres and Kronegger, 2019).

Taken together, the lessons learned from these selected examples of research at the University of Ljubljana in the past two decades are that only the integrative biology approach can produce multifaceted results, beyond the technology and knowledge silos in life sciences, in our view. This transdisciplinary approach offered by integrative biology allowed us to tackle big challenges in animal science of 21st century that in effect can usefully inform human and planetary health when viewed from a lens of One Health. Transdisciplinary and multispecies life sciences research using high-throughput multiomics technology platforms serve health scholarship well especially when they also include critical social sciences and humanities research to establish linkages between the contents and context of science.

Research-based teaching is an effective approach to improving the quality of student learning. For this reason, teaching models at the University of Ljubljana, Biotechnical Faculty, Department of Animal Science, are constantly adapted to allow for integrative research. To the extent that “context is everything,” such transdisciplinary thinking can help unpack complex questions in animal health and welfare, translation of discovery science to human and ecosystem health, environmental protection, pressing local and global societal needs, and sustainability along the food chain.
